# Using the Ferret as an Animal Model for Investigating Influenza Antiviral Effectiveness

**DOI:** 10.3389/fmicb.2016.00080

**Published:** 2016-02-04

**Authors:** Ding Y. Oh, Aeron C. Hurt

**Affiliations:** ^1^WHO Collaborating Centre for Reference and Research on Influenza, Victorian Infectious Diseases Reference Laboratory, Peter Doherty Institute for Infection and Immunity, MelbourneVIC, Australia; ^2^School of Applied and Biomedical Sciences, Federation University Australia, GippslandVIC, Australia; ^3^Melbourne School of Population and Global Health, University of Melbourne, ParkvilleVIC, Australia

**Keywords:** ferret, antiviral, animal model, influenza, effectiveness

## Abstract

The concern of the emergence of a pandemic influenza virus has sparked an increased effort toward the development and testing of novel influenza antivirals. Central to this is the animal model of influenza infection, which has played an important role in understanding treatment effectiveness and the effect of antivirals on host immune responses. Among the different animal models of influenza, ferrets can be considered the most suitable for antiviral studies as they display most of the human-like symptoms following influenza infections, they can be infected with human influenza virus without prior viral adaptation and have the ability to transmit influenza virus efficiently between one another. However, an accurate assessment of the effectiveness of an antiviral treatment in ferrets is dependent on three major experimental considerations encompassing firstly, the volume and titer of virus, and the route of viral inoculation. Secondly, the route and dose of drug administration, and lastly, the different methods used to assess clinical symptoms, viral shedding kinetics and host immune responses in the ferrets. A good understanding of these areas is necessary to achieve data that can accurately inform the human use of influenza antivirals. In this review, we discuss the current progress and the challenges faced in these three major areas when using the ferret model to measure influenza antiviral effectiveness.

## Influenza – the Disease

Influenza is a highly contagious respiratory disease causing symptoms ranging from headache, myalgia, malaise, sore throat, sneezing, and nasal discharge (Cox and Subbarao, 1999). Influenza virus is transmitted via virus-laden secretions propelled by coughing or sneezing from an infected person. Most influenza infections are self-limiting, lasting for one to 5 days but host factors such as age, pregnancy, and underlying medical conditions can increase the severity of illness (Gavin and Thomson, 2003). Influenza causes high global mortality and morbidity annually, with United States alone experiencing approximately 95,000–172,000 hospitalizations and 21,000–41,000 deaths annually (Lowen et al., 2006). The morbidity associated with seasonal influenza has a significant economic impact due to work absenteeism and puts huge pressure on the public health system.

## Influenza Antivirals

To date, the neuraminidase inhibitors (NAIs) are the only licensed class of antiviral drugs effective against currently circulating influenza viruses. Zanamivir (Relenza^TM^) and oseltamivir (Tamiflu^TM^) have been licensed since 1999 while newer NAIs, such as peramivir (Rapivab^TM^) and laninamivir (Inavir^TM^), are approved in Japan, and in the case of peramivir also in South Korea, USA, and China (Ikematsu and Kawai, 2011; Chairat et al., 2013; ChinaBioToday, 2013). However, the therapeutic and prophylactic efficacy of NAIs against ‘seasonal’ influenza infection remains hotly debated (Jefferson et al., 2014). The continuous risks posed by the emergence of NAI-resistant viruses (Takashita et al., 2015) and the pandemic potential of avian influenza viruses, such as A(H5N1) (Nguyen et al., 2013) and A(H7N9) (Hu et al., 2013), has sparked a major effort to develop new antivirals for human use.

**FIGURE 1:**
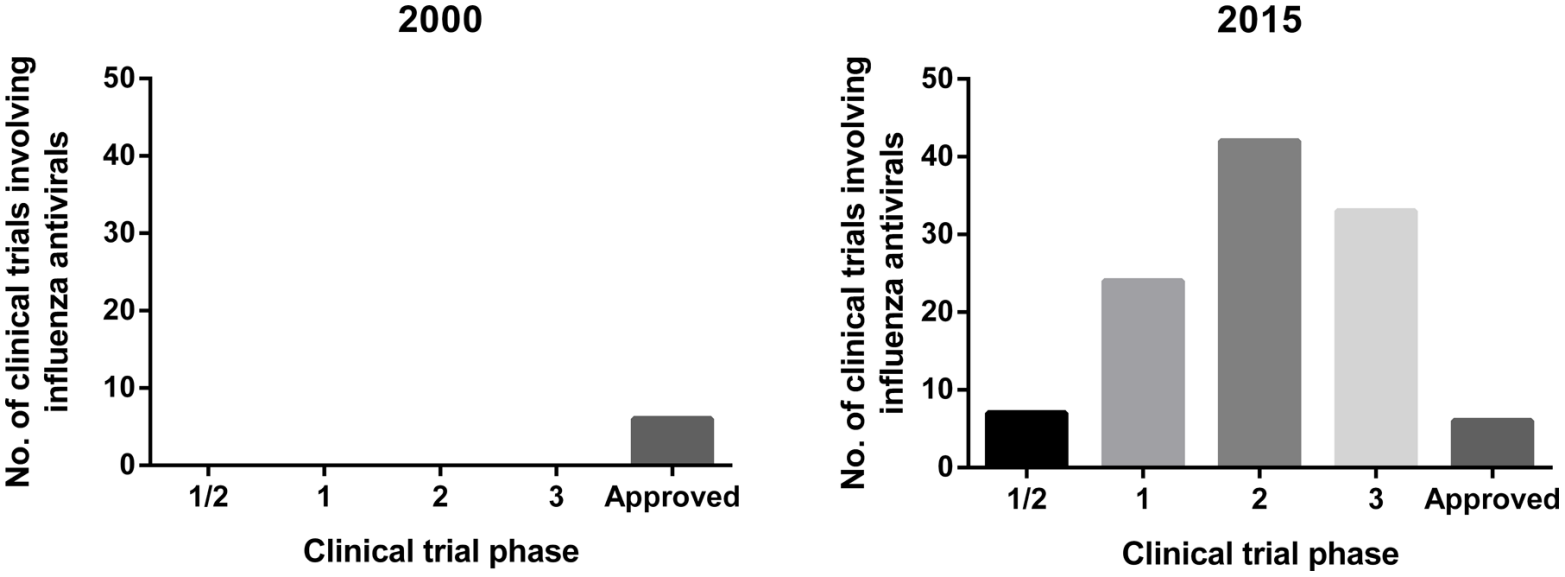
**Overview of clinical trials of influenza antivirals in year 2000 and 2015.** Data for 2015 extracted from clinicaltrials.gov (ClinicalTrials, 2015) using search terms: ‘Influenza’ and ‘antivirals’ and ‘antivirals treatment’.

Typically, investigational antivirals will first undergo *in vitro* efficacy screening, followed by *in vivo* testing in animal models to look at pharmacokinetics/pharmacodynamics (PK/PD), drug toxicity and drug effectiveness prior to clinical trials. As such, the choice of the animal model for assessing the effectiveness of these influenza antivirals becomes critical as it provides pre-clinical data that can inform the decision for progression toward clinical trials. Currently, there are a large number of influenza antivirals undergoing clinical trials, a substantial increase from the limited trials in 2000 (**Figure 1**). In the majority of human clinical trials of influenza antivirals, the primary endpoint used to assess the drug efficacy is the time to alleviation of clinical symptoms, such as cough, fever, sore throat, myalgia, lethargy, nasal congestion, and headaches, whereas other aspects, including the ability to reduce viral shedding, are considered secondary endpoints (Hayden et al., 1997; The MIST, 1998; Makela et al., 2000; Nicholson et al., 2000; Treanor et al., 2000; Haffizulla et al., 2014).

## Animal Models in Influenza Research

Animal models of influenza infection have played an important role in the understanding of viral pathogenicity and have served as pre-clinical models for the evaluation of vaccine candidates and new therapeutics (Kiso et al., 2010; Margine and Krammer, 2014; Marjuki et al., 2014). To date, there are many different animal models of influenza infection, namely ferrets, mice, guinea pigs, swine, non-human primates (NHP), and more recently, zebrafish (Gabor et al., 2014). The pros and cons of the different animal models of influenza to investigate disease pathogenesis, transmission, and vaccine development have been well-described in several published reviews and are summarized here in **Table 1** (Bouvier and Lowen, 2010; Lowen et al., 2014; Margine and Krammer, 2014; Thangavel and Bouvier, 2014; Davis et al., 2015; Enkirch and von Messling, 2015).

**Table 1:** Comparison of different animal models for influenza infection.

Clinical symptoms	Human	Animal model of influenza infection					
		Ferrets	Mice	Guinea Pigs	Swine	NHP	Zebrafish
Sneezing	Yes	Yes	No	No	Yes	Not always^a^	No
Nasal discharge	Yes	Yes	No	No	Not always^a^	Not always^a^	No
Lethargy	Yes	Yes	Yes	No	No	Not always^a^	Unknown
Fever	Yes	Yes	No	No	Not always^a^	Not always^a^	No
Weight loss	Yes	Yes	Yes	No	Minor	Not always^a^	No
Viral shedding	Yes	Yes	Yes	Yes	Yes	Yes	Yes
Experimental cost	-	Moderate	Low	Moderate	High	High	Low
Transmission between animals	-	Good	Poor	Good	Good	Poor	Unknown
Can infect with human influenza viruses?	-	Yes	No^b^	Yes	Yes	Yes	Unknown
A(H1N1)pdm09^c^	-	Yes	Yes	Yes	Yes	Yes	Not reported
A(H3N2)^c^	-	Yes	Yes	Yes	Yes	Yes	Yes
B^c^	-	Yes	Yes	Not reported	Yes	Yes	Not reported
Avian origin^c^	-	Yes	Yes	Yes	Not reported	Yes	Not reported

*NHP, Non-human primates. ^a^Clinical symptoms vary with different influenza strains. ^b^Serial passaging is required to ‘adapt’ the virus to replicate in majority of mouse strains. ^c^Influenza virus strain/subtype that have been tested in the animal model.*

### Animal Models in Influenza Antiviral Studies

Among all animal experimental models, mice are most commonly used for testing influenza antivirals mainly due to factors, such as lower experimental cost, ease of animal handling and the ability to use large numbers of animals to attain statistical power in a single experiment (Ryan et al., 1994; Mendel et al., 1998; Triana-Baltzer et al., 2009; Kiso et al., 2010; Bantia et al., 2011; Smee et al., 2012b; Zarogiannis et al., 2012; Marjuki et al., 2014). To date, weight loss, mortality (lethal model) and virus titer are the commonly used determinants of antiviral drug effectiveness in mice studies. Although these measurements are informative, the usefulness of mice in antiviral studies has been largely limited by the lack of clinical symptoms following influenza infection. The absence of clinical symptoms such as fever, sneezing, nasal discharge, and nasal inflammation in mice following influenza infection limits the extrapolation of mouse data to the human scenario where alleviation of symptoms are considered as the primary endpoint in clinical trials (**Table 1**). In contrast, the ferret is the only animal model which displays comparable clinical symptoms to that of humans following influenza infection (**Table 1**). In view of these factors, in this review we will discuss the current progress, limitations and the future directions of using ferrets to assess antiviral effectiveness against influenza infections.

### Ferret

Since the discovery of the susceptibility of ferrets (*Mustela putorius furo*) to influenza virus in the 1930’s (Smith et al., 1933), they remain one of the best animal models of influenza infection as they exhibit many of the clinical symptoms observed in humans following influenza infection, can be directly infected with human influenza virus without prior viral adaptation, and have the ability to transmit influenza virus efficiently between one another (**Table 1**). The susceptibility of ferrets to human influenza viruses is due to the presence of α2-6-linked terminal *N*-acetylneuraminic sialic acids (Neu5AC) in their respiratory tract which facilitates virus binding and the initiation of viral replication (Jia et al., 2014; Ng et al., 2014).

However, the use of ferrets for influenza studies has been limited by factors such as animal availability, genetic heterogeneity (out-bred) (Margine and Krammer, 2014), the requirement of a complex husbandry facility and caging system (Mabry et al., 2013; Enkirch and von Messling, 2015), and a lack of immunological reagents and genetically modified mutants for immunological investigation (Margine and Krammer, 2014; Enkirch and von Messling, 2015). As a consequence, ferret experiments can be limited by small sample sizes (*n* ≤ 5) (Belser et al., 2013b; Nishiura et al., 2013; Buhnerkempe et al., 2015), where large animal-to-animal variability has resulted in the detection of non-statistically significant trends of antiviral effectiveness between the treatment groups in variables, such as weight, temperature, nasal inflammation, and virus titer (Rowe et al., 2010; Govorkova et al., 2011; Oh et al., 2014, 2015). Ideally, a larger number of ferrets should be used but limitations, such as high experimental cost, low animal availability, limited caging capacity and ethical constraint, typically restricts most studies to group sizes of five or less ferrets. Unlike the more commonly used animal models, such as rodents and guinea pigs, the use of larger animals for experimentation, such as ferrets, can be met with greater scrutiny by animal ethics governing bodies on aspects, such as the choice of animal, animal numbers and husbandry concerns. Only a small number of countries or regions (such as USA, UK, and Europe) have guidelines regarding the husbandry of ferrets for animal experimentation. Therefore in the absence of any local directives, animal ethics committees may rely on guidelines provided by other countries, particularly where the committee has little experience in the use of ferrets.

**FIGURE 2:**
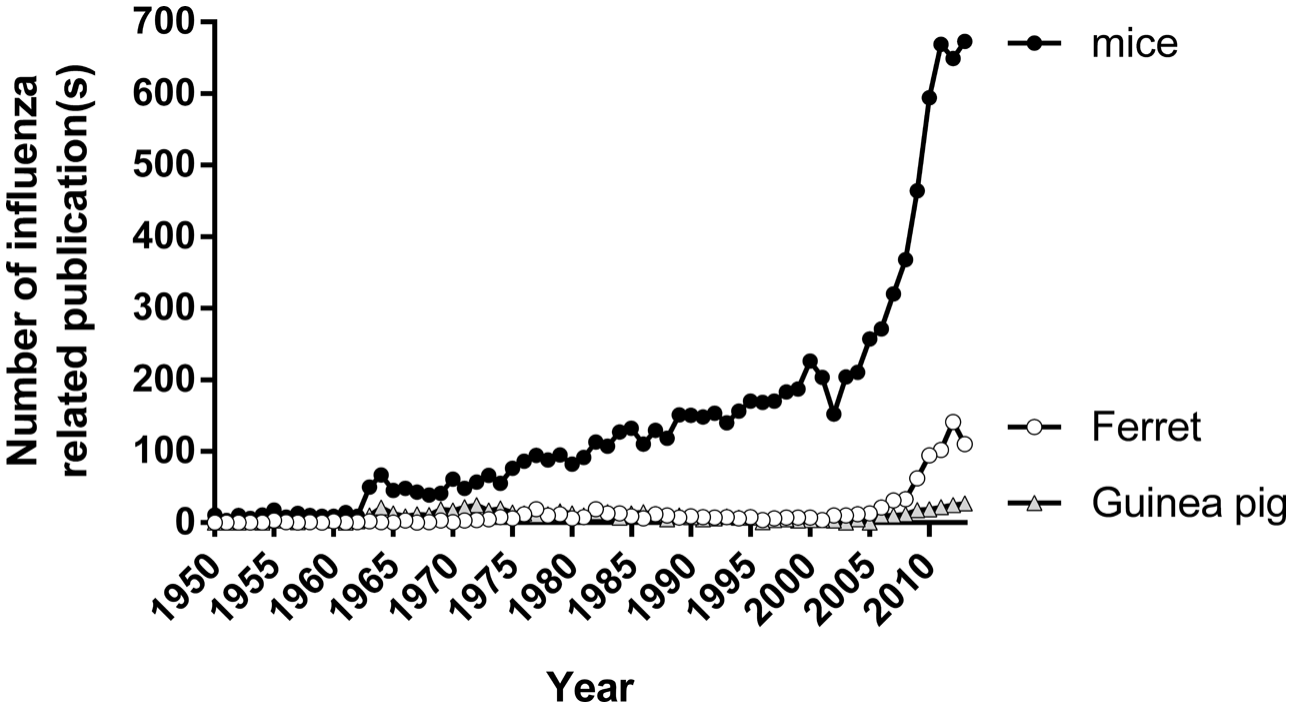
**The number of publications using different animal models of influenza.** Number of papers published on topics relating to influenza and mice/ferret/guinea pig from 1950 to 2013. Data tabulated by online automated yearly statistics of PubMed results (http://dan.corlan.net/medline-trend.html). Search terms used are ‘Influenza’ and ‘Mice/ferret/guinea pig.’

Despite these limitations, the use of ferrets in influenza research has increased considerably since 2008 (**Figure 2**). This increment was largely attributed to a global effort to better understand viruses with pandemic potential such as the avian influenza viruses A(H5N1) and A(H7N9), and the virus responsible for the 2009 pandemic, the A(H1N1)pdm09 virus (Govorkova et al., 2005; Yen et al., 2007; Boltz et al., 2008; Itoh et al., 2009; Maines et al., 2009; Munster et al., 2009; Belser et al., 2013a; Richard et al., 2013; Zhang et al., 2013). With the recent publication of the ferret genome (Peng et al., 2014), it is likely that the use of the ferret as a model of influenza will continue to rise.

To date, the ferret model has been used to investigate viral susceptibility and transmission (Yen et al., 2007; Maines et al., 2009; Zhang et al., 2013), and as a preclinical model to investigate poorly understood areas such as immunological responses to influenza in young children (newly weaned ferrets) (Huang et al., 2012), impaired immunity in older individuals (aged ferrets) (Paquette et al., 2014) and the treatment effectiveness of different antivirals to reduce influenza infection (Boltz et al., 2008; Kubo et al., 2010; Govorkova et al., 2011; Kitano et al., 2011; Marriott et al., 2014). Recently, the use of ferrets in influenza antiviral studies has extended to treatment effectiveness in an immunocompromised setting (van der Vries et al., 2013), the effectiveness of antiviral treatment or prophylaxis in preventing infections in secondary contacts (Oh et al., 2014) and the transmission of influenza between an infant and mother during breast-feeding (Paquette et al., 2015).

**FIGURE 3:**
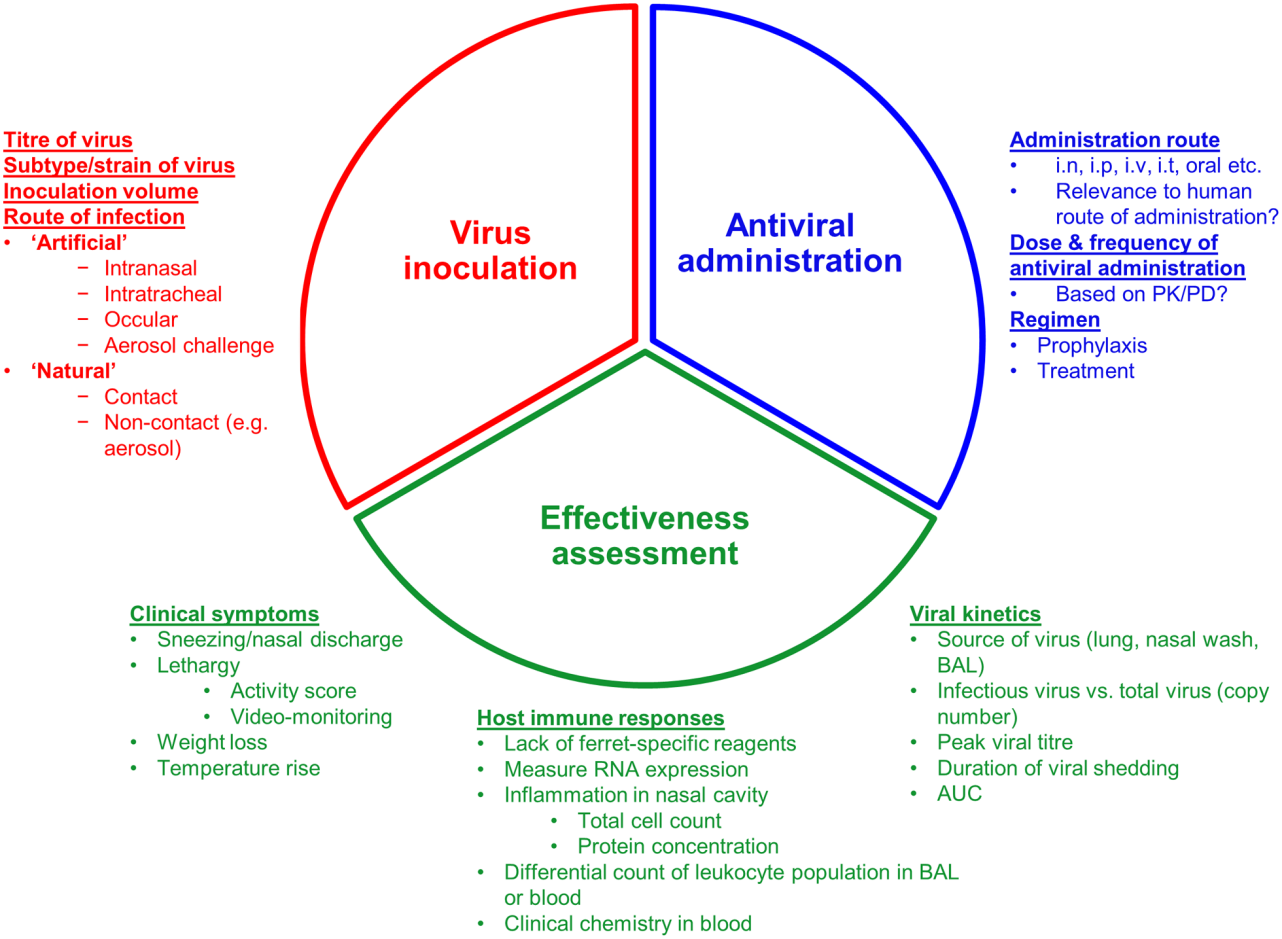
**Overview of the three experimental considerations when assessing an antiviral in a ferret model of influenza infection.** i.n: Intranasal; i.p, intraperitoneal; i.v, intravenous; i.t, intratracheal; PK/PD, pharmacokinetics/pharmacodynamics; BAL, bronchoalveolar lavage; AUC, area under curve.

There are a large number of experimental variables in a typical ferret antiviral effectiveness study that can alter the study outcome and that should be carefully considered to ensure that the most reliable data is generated. These variables are discussed in detail below and are summarized in **Figure 3**.

## Inoculation of Influenza Virus in Ferrets

The initiation of influenza infection in animal experimental models, and even in the human challenge model (Darton et al., 2015), is routinely carried out by instilling virus via the intranasal route. In general, intranasal instillation of virus is considered to be a simple procedure (Turner et al., 2011) compared to intratracheal (Kreijtz et al., 2013; van der Vries et al., 2013) or ocular (Belser et al., 2014), by which ferrets can also be infected in a reproducible manner. However, the decision on which inoculation route to initiate infection can be dependent on the virus strain to be tested. For avian influenza viruses, such as A(H5N1) and A(H7N9), intratracheal instillation is preferred over direct inoculation into the lower respiratory tract of ferrets, as it is found to induce a more severe pneumonia that more closely resembles pathogenesis observed in humans, compared to intranasal inoculation which causes mild to moderate pneumonia in ferrets (Bodewes et al., 2011; Kreijtz et al., 2013). For human seasonal viruses, such as influenza A(H1N1)pdm09, A(H3N2), and B viruses, intratrachel instillation has been used in only a small number of studies (van der Vries et al., 2013). Instead, ferrets can be effectively infected by intranasal instillation (Huang et al., 2011). However, the clinical severity of influenza infection via intranasal instillation is highly dependent on the volume of inoculum (Bodewes et al., 2011; Moore et al., 2014). A larger volume of inoculum (1 mL) results in the delivery of virus down into lower respiratory tract and into the lungs compared to smaller volumes (0.2 mL and 0.5 mL) which are primarily retained in the upper respiratory tract (Moore et al., 2014). As a result, ferrets intranasally infected with the larger volumes of inoculum have a more severe illness and lung histopathology compared to those infected with a smaller volume (0.2 mL) even when the same infectious titer of virus was used (Moore et al., 2014). These finding not only underscored influenza strains, inoculum route and volume to be important considerations in the ferret model of influenza infection but also highlighted the difficulties in interpreting data across studies using different inoculation protocols.

**FIGURE 4:**
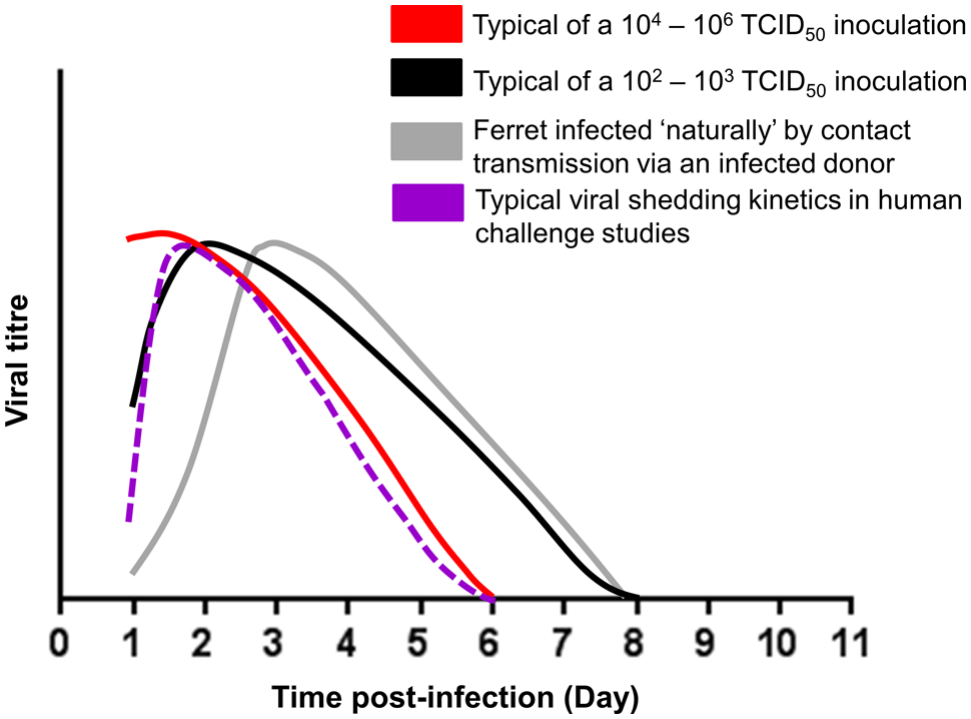
**Schematic representation of the viral shedding kinetics of intranasally infected (10^2^–10^3^, 10^4^–10^6^ TCID_50_ viral inoculum) ferrets, ferrets naturally infected by contact transmission via an infected donor and intranasally infected human in a challenge model (Hayden et al., 1999; Baccam et al., 2006).** Ferret viral data were adapted from published studies (McBrayer et al., 2010; Smith et al., 2011; Stark et al., 2013; Oh et al., 2014, 2015).

The alternatives to intranasal infection are the use of a ‘natural’ infection, that can be achieved by either contact (Roberts et al., 2012; Oh et al., 2014) or non-contact transmission (Hamelin et al., 2011; van der Vries et al., 2011; Herfst et al., 2012) of virus by exposing a naïve ferret to an infected ferret in the same or adjacent cage, or aerosolized challenge (Gustin et al., 2011). Despite ‘natural infection’ being a more clinically relevant route of transmission, few studies have used this methodology, presumably due to the additional experimental time involved, additional ferret use, the requirement for larger cages to house multiple animals for contact transmission or a more complex caging system for non-contact transmission, and the lack of transmissibility of influenza viruses, such as avian influenza A(H5N1) and A(H7N9) (Yen et al., 2007; Belser et al., 2013a) and influenza B virus (Kim et al., 2015), via the aerosol and/or contact route. Nevertheless, ‘natural’ infection has the clear benefit of infecting ferrets with a realistic infectious viral dose and results in viral replication kinetics that better mimic those of ‘natural’ influenza infections in humans (**Figure 4**). Unlike ‘natural’ infection, which relies on infected donor ferrets to transmit viruses to naïve recipients, a more standardized alternative is aerosol challenge which involves exposing naïve ferrets directly to known doses of aerosolized influenza viruses delivered by specific devices. Ferrets exposed to low aerosolized viral dose (1.5–2.2 FID_50_) have been shown to exhibit similar viral shedding kinetics and disease pathogenesis to those infected via ‘natural’ infection (Gustin et al., 2011). In contrast to ‘natural’ infection, aerosol challenges eliminates the need for donor ferrets, allows ferrets to be infected with viruses with poor aerosol transmissibility, and enables the manipulation of variables, such as dose and timing of viral exposure, to achieve a more standardized way of infecting ferrets in a ‘natural’ fashion. However, few studies have reported using this methodology presumably due to the high cost involved in requiring specialized aerosol system and cages for such inoculation protocol.

The majority of ferret infection studies have used intranasal infections with inocula of high infectious viral titers (e.g., 10^6^ TCID_50_/PFU/EID_50_ per animal) which may result in a very large number of infectious particles infecting the nasal epithelial in a short time period (Maines et al., 2009; Govorkova et al., 2011; Kim et al., 2013; Stark et al., 2013; Paquette et al., 2014; Marjuki et al., 2015). Ferrets infected with high viral inocula (10^4-6^ TCID_50_/animal) can show viral shedding kinetics where titers peak as early as 1 day post-infection (**Figure 4**). In contrast, ferrets infected with a lower viral inocula (10^2-3^ TCID_50_/animal) can show a more ‘typical’ viral shedding curve that more closely resembles a natural infection where viral titers gradually rise, peak and then fall (Baccam et al., 2006; **Figure 4**). Although high titer viral inoculums have been used to increase the chances of infection or increase pathogenicity, several studies have shown that the initial titer of the inoculum does not correlate to disease severity in ferrets (Marriott et al., 2014; Oh et al., 2015).

Influenza antivirals, such as NAIs, typically act by interrupting the viral replication cycle, whereas antibiotics directly eliminate and reduce the causative pathogen (McCullers, 2011). Therefore, the common practice of infecting ferrets with a high viral inoculum could overwhelm the host (ferret) with an unrealistically large number of infectious viral particles and under such experimental conditions, the effectiveness of an antiviral treatment could be undermined as the antivirals would not be able to contain such rapid onset of viral infection. As demonstrated by Marriott et al. (2014), oseltamivir treatment significantly lowered viral shedding, lowered inflammatory nasal cell count and improved activity levels following a 10^2^ PFU/animal dose of infection, but had no significant effect on these parameters when a high viral inoculum of 10^6^ PFU/animal was used (Marriott et al., 2014). Therefore, in the context of antiviral testing in ferrets and in particular to intranasal inoculation, careful consideration should be given to the amount of virus used, to prevent undermining the effectiveness of antivirals in influenza infection.

## Drug Administration

Administration of therapeutics into animals requires the careful consideration of many factors including the drug pharmacology, concentration, volume, timing, frequency of dose and route of administration (Urso et al., 2002). The antivirals for influenza treatment that are currently approved or in late-phase clinical trials in humans encompass various routes of administration including oral, inhaled and intravenous (**Table 2**). Where possible the route of administration in animals should follow the same route of delivery as in humans so that the results can be better extrapolated to the findings in man (Turner et al., 2011; **Table 2**).

**Table 2:** The differences in antiviral drug administration route between human and animal model of influenza infection.

Drugs	Approved administration route in human	Typical administration route used in animal studies		Reference
		Ferrets	Mice	
Amantadine	Oral	Intraperitoneal	Oral	Herlocher et al., 2003; Bantia et al., 2011; Smee et al., 2012a
Rimantadine	Oral	NR	Oral	Smee et al., 2012a
Oseltamivir	Oral	Oral	Oral	Govorkova et al., 2011; Marriott et al., 2014; Oh et al., 2014, 2015; Bird et al., 2015; Marois et al., 2015
Zanamivir	Powder inhalation	Intranasal (L)	Intranasal (L)/Intraperitoneal	Herlocher et al., 2003; Hurt et al., 2010; Smee et al., 2012a,b
Laninamivir	Powder inhalation	Intranasal (L)/Intratracheal (P)	Intranasal (L)	Kubo et al., 2010; Panozzo et al., 2015
Peramivir	Intravenous	Intravenous	Intravenous/Intramuscular/Oral	Yun et al., 2008; Bantia et al., 2011; Kitano et al., 2011; Smee et al., 2012a
T-705	Oral	NR	Oral	Kiso et al., 2010
DAS181^a^	Powder inhalation	NR	Intranasal (L)	Marjuki et al., 2014

*^a^Investigational drug; NR, Not reported; L, Liquid; P, Powder.*

The delivery of parenterally administered antivirals, such as peramivir, in ferrets is relatively straight-forward and can be easily administered by intravenous injection (Yun et al., 2008; Kitano et al., 2011). Administering drugs via the enteral (oral) route in ferrets can be limited by many factors such as the requirement for multiple dosing regimens, poor drug solubility and palatability of the drug to animals. In ferrets, direct feeding of orally administered compounds is preferred over oral gavage (which is commonly used in mice) as it does not require anesthesia, which can impact on the animal’s health and behavior if given repeatedly, such as twice daily for 5 days. For oseltamivir administration, ferrets can be fed oseltamivir phosphate dissolved in sugar solution to achieve an accurate dosing (Oh et al., 2014, 2015). The drug in sugar solution is well-tolerated by ferrets allowing multiple dosing without the need for sedation, although higher doses of the drug (e.g., 25 mg/kg) are less readily swallowed than the standard 5 mg/kg dose. In contrast, oral antivirals with poor solubility, such as nitazoxanide (Haffizulla et al., 2014; Rossignol, 2014), pose difficulties when administering to ferrets, and therefore alternative methods, such as spiking the drug into food or in special ‘treats’, may be necessary (Turner et al., 2011).

The delivery of inhaled drugs, such as zanamivir and laninamivir, to ferrets poses additional challenges. Both zanamivir and laninamivir are delivered to humans as a dry powder formulation that is actively inhaled by the patient via specially designed inhalers (Chairat et al., 2013). Mimicking this type of administration in animals is difficult, and is presumably why the majority of animal studies investigating zanamivir (Herlocher et al., 2003; Hurt et al., 2010; Smee et al., 2012a,b) and laninamivir (Kubo et al., 2010) have dissolved the compounds in saline or water and administered intranasally (**Table 2**). While intranasal instillation of drugs is a simple technique that can be easily adopted by most laboratories, the relevance of delivering the drug in this manner when it is designed to be inhaled is questionable (Turner et al., 2011). In addition, it has been demonstrated in mice that intranasal instillation of drug administration can exacerbate viral infection leading to lower drug effectiveness (Smee et al., 2012b). Insuﬄators are devices that have been widely used as a non-invasive method to administer powdered drugs for drug deposition studies in small animals such as mice or rats (Nahar et al., 2013), but have been less commonly used in larger animals such as ferrets. To bridge this unmet need for a delivery system for powdered antivirals such as laninamivir, we recently characterized the usage of a dry powder insuﬄator to deliver laninamivir octanoate (LO) to ferrets prior to influenza infection (Panozzo et al., 2015). *In vitro* laser diffraction analysis showed that ∼80% LO together with its lactose carrier can be effectively discharged from the device and intratracheal administration of LO in anesthetized ferrets can be easily performed (Panozzo et al., 2015). For LO where a single dose is sufficient, the method is highly applicable, but repeated dosing would be difficult due to the need for regular anesthesia. The use of the insuﬄator device may be useful to investigate other inhaled drugs in ferrets, such as DAS181.

## Drug Dosage

Besides route of administration, accurate evaluation of the effectiveness of antivirals in animals is also dependent on the dose of drug being administered. In humans, the optimal dose is determined by pharmacokinetic/pharmacodynamic (PK/PD) analysis. The PK/PD of influenza antivirals is often determined during pre-clinical testing in animals by pharmaceutical companies (Urso et al., 2002), but data from these studies is often not in the public domain. For example, the majority of studies have used 5 mg/kg oseltamivir phosphate in ferrets as being ‘equivalent’ to the 75 mg dose in humans, although limited publicly available PK/PD data is available to support this (Li et al., 1998; Mendel et al., 1998; Reddy et al., 2015). At the time of writing this review, we have found only a small number of PK studies of the influenza antivirals in animals such as mice, rats, and ferrets (**Table 3**). As the generation and analysis of PK/PD data can involve a large number of animals and requires specialized modeling expertise, it is not always a viable option for research laboratories to complete such studies. Therefore, if PK/PD data of approved drugs in ferrets or other animals was made publicly available in open access databases, then this would greatly assist and encourage the use of a consensus dose in an animal to allow a better assessment and comparison of the effectiveness of the drugs across different studies.

**Table 3:** Pharmacokinetics reports of different influenza antivirals in mice, rats, and ferrets.

Drugs	Mice/Rats	Ferrets
Amantadine	NR	NR
Rimantadine	Hoffman et al., 1988	NR
Oseltamivir	Li et al., 1998	Li et al., 1998; Reddy et al., 2015
Zanamivir	Li et al., 1998	NR
Laninamivir	Koyama et al., 2009, 2010	NR
Peramivir	Kodama et al., 2014	Kitano et al., 2011
T-705	NR	NR
DAS181	NR	NR

*NR, Not reported.*

## Parameters to Assess the Effectiveness of Antiviral Treatment

### Pathology

One advantage of using the ferret is the ability to track viral shedding kinetics from both the upper and/or lower respiratory tract via nasal washing or lower bronchoalveolar lavage (BAL), although the latter is rarely used (Lee et al., 2014). In addition, ferrets can be monitored for clinical symptoms associated with human influenza infections including weight loss, rise in body temperature, sneezing, nasal discharge, and lethargy (**Table 4**). Whilst weight, temperature and symptoms such as sneezing and nasal discharge can be easily assessed, lethargy is more challenging to quantitate accurately. Conventionally, the activity of ferrets has been determined by visually assessing movement of the animal and assigning an arbitrary score (Reuman et al., 1989; Govorkova et al., 2011; Kim et al., 2013; Stark et al., 2013; Marriott et al., 2014; Oh et al., 2014). However, we have recently reported on the use of video-tracking methodology, which allows the activity level of ferrets to be quantitated by computer software analysis (Oh et al., 2015). Not only did video-tracking improve the sensitivity of detecting activity changes post-infection, it also enabled the assessment process to be simplified and less prone to bias. To date, many studies including ours have shown that treatment with an antiviral, such as oseltamivir, can improve the activity level of ferrets infected with influenza (Govorkova et al., 2011; Marriott et al., 2014; Oh et al., 2014, 2015). This improved methodology allows a more accurate assessment and extrapolation of the associated effect of antivirals on activity or ‘wellness’.

**Table 4:** Different types of clinical and symptomatic parameters measured in influenza studies in ferrets.

Parameters		Reference
Symptomatic	Sneezing	Huang et al., 2011, 2012; Roberts et al., 2012; Kim et al., 2013; Paquette et al., 2014
	Weight loss	Govorkova et al., 2011; Huang et al., 2011, 2012; Kim et al., 2013; van der Vries et al., 2013; Marriott et al., 2014; Oh et al., 2014, 2015; Paquette et al., 2014; Panozzo et al., 2015
	Body temperature	Govorkova et al., 2011; Huang et al., 2011; Roberts et al., 2012; Kim et al., 2013; van der Vries et al., 2013; Marriott et al., 2014; Oh et al., 2014, 2015; Panozzo et al., 2015
	Activity (manual scoring)	Govorkova et al., 2011; Huang et al., 2011; Kim et al., 2013; Marriott et al., 2014; Oh et al., 2014
	Activity (video-tracking)	Oh et al., 2015
Virological	Viral shedding kinetics^a,b,c^	Govorkova et al., 2011; Huang et al., 2012; Kim et al., 2013; van der Vries et al., 2013; Marriott et al., 2014; Oh et al., 2014, 2015; Paquette et al., 2014; Panozzo et al., 2015
Histopathological	Lung histology	Cameron et al., 2008; Rowe et al., 2010; Huang et al., 2011, 2012; Kim et al., 2013
Inflammation	Total viable cell count^a^	Govorkova et al., 2011; Marriott et al., 2014; Oh et al., 2014, 2015; Panozzo et al., 2015
	Total protein concentration^a^	Govorkova et al., 2011; Oh et al., 2014, 2015; Panozzo et al., 2015
Immunological	Differential cell count (innate immune cells)^c^	Kim et al., 2013
	Influenza-specific antibodies	van der Vries et al., 2013; Oh et al., 2014, 2015; Panozzo et al., 2015
	Hematology chemistry	Stark et al., 2013
	RNA expression (RT-PCR)	Kim et al., 2013; Carolan et al., 2014, 2015; Paquette et al., 2014
	RNA expression (Microarray)	Cameron et al., 2008; Rowe et al., 2010

*^a^Nasal wash; ^b^Assay by TCID_*50*_, PFU or RT-PCR; ^c^Bronchoalveolar lavage.*

### Immunology

The major disadvantage of using ferrets as a model for studying influenza (or influenza antiviral effectiveness) is the lack of validated ferret-specific reagents, such as antibodies, to study the immunology of disease pathogenesis. However, because of the significant benefits of the model many laboratories are now attempting to validate cross-reactive antibodies and develop novel reagents for ferret research (Rutigliano et al., 2008; Martel and Aasted, 2009; Music et al., 2014). One such collaborative effort is chaired by the United States Centre of Excellence for Influenza Research and Surveillance group at St. Jude Children’s Research Hospital in Memphis, USA. To date, several groups have assessed ferret host immune responses to influenza infection based on RNA expression by either quantitative real-time PCR in either nasal washes, blood or lung (Maines et al., 2012; Carolan et al., 2014, 2015; Paquette et al., 2014; Vidana et al., 2014), or by using a canine microarray platform (Cameron et al., 2008; Rowe et al., 2010; **Table 4**). Recently, the report of an annotated ferret transcriptome during influenza infection has also opened up an avenue for interrogating the global host gene expression to further understand host immunity toward influenza infection (Leon et al., 2013). In addition, simple immunological analysis such as measuring total cell count or protein concentration in nasal wash samples can also be useful as a determinant of inflammation (Govorkova et al., 2007, 2011; Marriott et al., 2014; Oh et al., 2015; Panozzo et al., 2015). Other basic immune measurements include differential staining of various leukocytes populations in the BAL or blood, and also clinical chemistry of different proteins in blood post influenza infection (Kim et al., 2013; Stark et al., 2013; **Table 4**).

Despite a great effort to study the interplay between influenza infection and immunity in ferrets, the effect of antivirals on immunity in this model is still poorly understood. To date, the majority of the immunity-related studies on influenza antivirals have used mice as the animal model (Burger et al., 2000; Wong et al., 2011; Bird et al., 2015; Marois et al., 2015). Oseltamivir was found to reduce innate cell populations in BAL, such as neutrophils and macrophages, when given prophylactically, and reduced pro-inflammatory cytokines when given pre- and post-infection (Wong et al., 2011). In contrast, the effect of oseltamivir on the adaptive immune cell population, such as cytotoxic CD8^+^ T cells, is still unclear. While Burger et al. (2000) reported that oseltamivir had minimal impact on the splenic population of T and B cells, and had no effect on the cytolytic activity of CD8^+^ T cells and natural killer cells in A(H1N1) influenza-infected mice (Burger et al., 2000), a recent study by Marois et al. (2015), found oseltamivir reduced circulating and tissue-resident effector and memory CD8^+^ T cells in A(H1N1) influenza-infected mice and significantly reduced the protective effect in a subsequent infection episode (Marois et al., 2015). In addition, a study by Bird et al. (2015) showed that although oseltamivir treatment reduced the number of CD8^+^ T cells in mice, they had normal recall responses and conferred protection against subsequent infection (Bird et al., 2015).

## Future Directions for the Use of Ferrets to Assess Antiviral Effectiveness

To date, the ferret remains as one of the preferred models for assessing influenza infection and as such has become an important model for antiviral testing. Although the ferret poses many advantages compared to other animal models, this review has highlighted some of the challenges of using the ferret to assess antiviral effectiveness. Researchers conducting studies in the ferret model of influenza infection need to consider progressing toward a more ‘natural’ infection methodology by either contact or non-contact transmission, or aerosol challenge, using drug doses that are based on PK/PD analysis and to deliver the drugs via a relevant route (**Figure 3**). The development of more robust parameters to measure antiviral effectiveness in ferrets, such as computational analysis of behavior/activity and the development of ferret-specific reagents to explore the immunological effects of antiviral treatment, will both greatly improve our understanding of antiviral modes of action and the effect on viral pathogenesis. In our opinion, the application of ferrets in antiviral studies should not be limited to just understanding therapeutic effectiveness. Instead, the ferret model can be applied to address other questions such as the *in vivo* effectiveness of antivirals against drug ‘resistant’ viruses, and the effectiveness of different antiviral treatment or prophylaxis strategies on preventing or minimizing transmission. Therefore, we anticipate that with continuous development and refinement of the ferret model for influenza antiviral testing, it has the potential to provide more meaningful data to better inform human use of influenza antivirals.

## Author Contributions

Both authors made substantial, direct and intellectual contribution to the work, and approved it for publication.

## Conflict of Interest Statement

The authors declare that the research was conducted in the absence of any commercial or financial relationships that could be construed as a potential conflict of interest.
